# The Psychology of Epochs
*Cosmos. By Humboldt.


**Published:** 1852-04-01

**Authors:** 


### Art. II.—THE PSYCHOLOGY OF EPOCHS.*

The chart of universal history displays a succession of prominent events, which have overwhelmed and changed the face of the political, as completely as the great deluge did that of the physical globe. These events form a portion of that development of the expanded intellect scattered over the space of centuries. Each crisis in this mighty series has been as decisive as it was transient; like the gourd of the prophet, it arose in a night and perished in a night; and the troubled morning that afterwards broke on the astonished world was entirely new and unexpected.

The human species, like the individual, is, in its main elements, everywhere the same. East, west, north and south, communicate with each other; the ancients with the moderns, the young with the old,

* Cosmos. By Humboldt.

and, by the help of revelation, to-day with futurity. Identity is the common quality of our nature; nor is it until we examine the person by himself, or nations in the mass, that we recognise the essential difference that distinguishes their respective conditions, ages, and epochs. The laws under which we dwell, the climate in which we are born, the number of years that we have lived, or that the earth has existed; our natural complexions, infirmities, habits, and propensities; create such absolute discrepancies of colour, form, features, and expression, that, when brought into juxta-position with each other, we can hardly believe ourselves to be the children of one large family sprung from the same root and common stock of all. Look back on the past with an impartial eye; disperse the halo of classic light that surrounds each object with a fictitious splendour; and candidly examine the psychology of bygone ages in the field of a microscope illuminated with the broad rays of criticism and truth. Stand forth, O ye generations long since extinct, and pass by unveiled before us in your own solemn grandeur and stateliness of thought and passion!

Fifteen hundred years ago, there existed, or rather subsisted, below the horizon of the barbarous and civilized populations of the earth, a vast body of human beings without a recognised rank or title, *vulgus sine nomine*, grovelling on their knees, and supporting the huge fabric of society upon their degraded and crouching shoulders. We shudder at the thought of an abject set of mortals destitute of poetry, law, and right, speechless and passive. They were neither human beings nor things; yet they were both a thing and a human being without which the old world could not have held itself together for a single day, or hour,—the necessary, but invisible pivot upon which turned the heartless paganism of three thousand years. It belonged not to any city or province in particular, nor to any one quarter of the globe, more than to all the rest. It was a common domestic commodity in daily use, from east to west. Persepolis, Athens, and Heliopolis, so different from each other in every other respect, were exactly the same in this, that slavery was a piece of state machinery successfully practised by them all. Empires and republics, leagues and institutions, rose up, flourished for a while, and vanished from the face of the earth, like successive crops of vegetation, while slavery remained the same beneath every change, an indigenous weed deeply rooted in every soil. Time, that ameliorates most things else, only helped to lock the fetters still more tightly round the wrists of the sulky slave. The reigning world stalked by and trod upon his neck. The pomp of power banished him from the rites of religion and the sacred service of the gods, as sternly as it repulsed him from the frowning portals of the great. The only boon that pride assigned him for his bitter portion, was that narrow and undisputed isthmus of mortality, just lying between life and death.

At this distance of time, and circumstanced as we now are, this gloomy description appears incredible. It is more like a pathetic episode in some romantic novel of the day, than a cold reality, which had an actual existence; neither is it easy to account for its origin and continuance. Gibbon, who is reluctant to admit of any statement adverse to the credit of heathenism, whose honour he undertook to vindicate, blinks the inquiry by ascribing it to the right of battle; and Montesquieu, who is sometimes superficial, ingeniously imputes it to the tyranny and enervating climate of the East. But neither of these explanations meets the point in question. For slavery formed as much a part of the vivacious confederacies of Greece, as it did of the monotonous despotisms of Asia; it prevailed in the cold regions of the north as much as it abounded in the warmer countries of the south. We may trace it everywhere, among the savage as well as among the more civilized populations;—indeed, it may be affirmed, that, wherever the foot of man pressed the ground, thither slavery accompanied or pursued him, to his lasting vexation and disgrace. Like Gibbon, Rousseau and Hobbes have sought for its cause in the result of arms; and the lawyers of the Justinian period derived its name from *servus de servatus,* a person reserved *ex prædâ victorum,* as the prize of victory. But this legal definition, although framed within sight of slavery when it was just beginning to be impugned and exploded, does not solve the difficulty. Even granting that it sprung from the right of conquest, it does not clear up the puzzling part of the inquiry, namely, how it could have been tolerated and maintained, without dispute or protest, for so many centuries in succession? No philosopher ever rose up to oppose it. Popular opinion was decidedly in its favour. It was based upon the common consent of the world, and insisted upon, not only without inflicting any violence on any preconceived prejudices, but directly in accordance with an acknowledged consecration of the principle of slavery itself. It was venerated as a right divine.

The slave was a human being the same as his owner, with the same passions, hopes, and fears, elicited, of course, in a different manner, owing to the same circumstances operating differently on them both. Their number was immense. During the civil commotions of the Gracchi, there was a servile war in Sicily, and 70,000 of them revolted at once. At Athens, in the time of Demosthenes, they were calculated at 400,000, which was three times as many as the free inhabitants of the city, the foreign settlers included. In the Peloponnesian war, 20,000 passed over to the enemy, as Thucydides tells us. The same author says, that at Chios their number was very considerable, and that their defection, when they deserted to the Athenians, reduced their masters to great extremities. At Rome, their multitude was such that they were afraid of giving them a distinctive dress, or uniform, lest it should make them acquainted with their own overwhelming force. Catiline might have succeeded in his conspiracy, had he but armed the slaves; only he might very reasonably have been afraid of their managing the victory so as to suit their own purposes instead of his. Alaric was determined in his resolution of sacking Rome by a re-inforcement of 40,000 slaves, who ran away from the city, and joined the ranks of the barbarians, for the sake of sharing in the expected plunder. At Tyre, the slaves once rose up in a body against their masters, and massacred them all. The Scythians, on their return from the Median war, found their slaves in rebellion, as Herodotus tells us, in Melpomene, and were obliged to abandon their country to them, or recovered it only after a very severe conflict. Cæsar, in his Commentaries, bears witness to their numbers in Gaul; and Tacitus says the Germans held them in great contempt, and counted their lives as nothing.

Slavery continued in England for a considerable time after the conversion of the Saxons. They were by far the most numerous class of the community. The words villagers, villici, villani, are derived from them.

The degradation in which they were held is incomprehensible to us in this period of the world. Herodotus says the Scythians deprived their slaves of sight on account of some disgusting office they had to perform in their household; and Plutarch mentions, in his "Life of Cato," that this famous man used to sell his old slaves at any price, to rid himself of the expensive burden of supporting them to the end of their days. It was customary to expose slaves who were sick and useless to perish miserably on an island of the Tiber. They were frequently employed in chains at the most laborious drudgery; and for trivial offences, and even on mere suspicion, were sometimes put to death under the most horrid tortures.

This terrific picture, which has no counterpart in modern society, might be enlarged without exaggeration. The slavery of our factory system, a complaint so justly urged at the present moment, is almost perfect freedom compared with that of the slave in pagan times. They were commodities for traffic and barter in the market-place, where they were exhibited for sale, and trotted out in the same manner as a jockey shows off the paces of his horse to the best advantage. They varied in price from ten to twenty pounds sterling and upwards. Plato, who was captured by pirates, was ransomed for about £112 of our money. Diogenes, who experienced the same misfortune, remained in bondage, and endeavoured to teach his master's children the meaning of happiness and virtue. Tacitus mentions 400 slaves who were put to death for not having prevented the assassination of their master. Caius Cassius pronounced an oration on the occasion, reported at length by the historian, in support of the motion for inflicting the penalty of death on these unfortunate creatures, and in opposition to those who pleaded their ignorance as an excuse—*plurimorum indubiam innocentiam miserantium.* This severe decision of the senate gave rise to a tumult, and Tacitus coolly remarks, that Cæsar was forced to line the way with troops as the condemned multitude of every age and sex were led to the place of execution. We can scarcely credit our senses at this recital, more especially when it is coupled with a piece of intelligence, like that of a newspaper report, that upon the house dividing on the question, the senators were found nearly unanimous in favour of carrying out the extreme penalty of the law—*nemo unus contra ire ausus est—prevaluit tamen pars quæ supplicium decernebat.* But life was estimated at a cheap rate in those times; for persons of property disposed of their slaves in the same manner as people of wealth now do of their farming stock, &c., on the sale of their estates; and Pliny, in his Natural History, quoted by Gibbon, mentions the instance of a freedman, in the reign of Augustus, who, although he had suffered great losses in the civil wars, yet left behind him 4116 slaves, included in the description of his *other* cattle, which were very numerous. It is only when viewed in this most humiliating light, that we are enabled to see the utterly abject condition of this ignominious class of—*animals,* shall we call them? or *men,* or *fellow-creatures ?*—or to appreciate at its inestimable value the divine magnanimity of one who came to emancipate or redeem them *all* by willingly assuming the form of a slave—*formam servi accipiens.* This was the practical drift and aim of St. Paul's letter in supplication for Onesimus, a run-away slave, liable to death on that account, had he been caught and brought back to his owner. Ecclesiastical history records, that Philemon, to whom the apostle had written on this delicate subject, pardoned and emancipated his disobedient domestic, and that Onesimus made so much progress in religion as to merit the episcopacy of Ephesus after Timothy. This elevation and distinction must have been very galling to the aristocratic pride of the Gentiles, who never deigned to treat a freedman much better than they were in the habit of treating their vile slaves;—*servile vulgus fuère,* are the words of Justin.

We may here pause, and inquire what was the mental effect of such a state of things on the masses of mankind?—in short, what was the psychology of so many ages in this respect? Nothing but the darkest passions could be engendered by an unmitigated tyranny of this awful kind. The first effect of contumely and scorn, apart from the irritation excited by personal restraint, is, upon a generous nature, the most bitter feeling of desolation and woe. Were not the heart made to pulsate safely with contrary emotions, this feeling is so deadly and intense, that there is no doubt it would, if long continued, wither the brain and destroy life. In the finest spirits, indeed, such is the case, as we learn from the calamities that every now and then take place within the range of our own observations; nor are the tales of madness and death in consequence of unrequited love, or unmerited contempt and desertion by those whom we esteem, and from whose countenance we expect to derive both sympathy and support, to be discarded as nursery rhymes and childish gossip. Unhappily, *we* know that very often they are but too real and too true. The admirable manner in which we are all united in one great family on earth, is the reason why we cannot sever a single link from the chain that binds us, without inflicting some serious injury in the attempt.

The natural independence of man rebukes him for yielding to useless regrets. Dashing away the tear that moistens his eye, and hiding the blush that mantles on his cheek, he smites his breast; and, glancing up to heaven for help, he buckles on the burden of his pack, and betakes him doggedly to his hateful toil. Revenge is the cherished passion of his breast—deep, settled, determined revenge. Selfishness springs from the instant necessity of self-preservation—profound selfishness, unsatiated self-love. Covetousness, that dismal vice, swells the veins, together with hatred, and obstinacy, and the spirit of insurrection, and desperate struggles to escape. Behold the fiend formed by public or private tyranny—the once noble-minded man transmuted, by an accredited system of penal enactments, into a conspirator, an outcast, a villain, and a slave! Do you want an example in proof of this allegation? Read the biography of the dastard Eutropius, the prime minister of the Emperor Arcadius, a slave of the lowest description, a fellow of an infamous character, purchased by an officer of the imperial guard, emancipated, and introduced into the palace, where, by cunning and hypocrisy, he contrived to gain the esteem of the great Theodosius. Abandoned by his friends, if ever he had any, and protected from the fury of the populace by St. Chrysostom, whose destruction he had already planned, he was banished, and at last beheaded. He was the personification of that pitiable form of humanity engendered by a heartless social system. The eunuch Narses, who, to suit his own purposes, re-conquered and betrayed Italy, in the reign of Justinian, was a prince in action compared with the despicable Eutropius. The bloody insurrection of 4000 slaves, under Herdonius, which struck terror into every family in Rome, is another instance of the dire malignity fomented by unrelenting oppression; and the revolt of Spartacus was so formidable and resolute, that its suppression demanded the presence of one of the ablest generals, and the valour of some of the choicest legions.*

It was a political difficulty which every legislator grappled with, but in vain. Emancipation would have overthrown the world, as, in effect, it did overthrow it, when the barbarians from the north enforced it. Plato regarded the slave as one deprived of half his mind. Homer was of the same opinion. Aristotle thought still more meanly of them.† Tacitus implies that they were entirely untrustworthy, which in one sense was certainly true. They were the subject of constant legislation; and laws, protective and coercive, were enacted to secure the master against his slave, and the slave against his owner. Lacedæmon, by vigorous measures, drove them to revolt. Athens, on the contrary, by gentler methods, made them insolent. As the Roman empire became matured, their condition was ameliorated. The youths of a promising character were instructed in the arts and sciences, and their price was ascertained by the degree of their skill and talent. Almost every profession, either liberal or mechanical, might be found in the household of an opulent senator. Many of the Roman physicians were slaves. Athenæus, quoted by Gibbon, asserts that some ostentatious Romans possessed as many as ten or twenty thousand slaves. A learned one sold for many hundred pounds sterling; and Atticus always bred and taught them himself. It was a freedman that preserved and edited Cicero's Letters. But under their most advantageous circumstances they were still in bondage.

If, however, the slave laboured under many evils almost intolerable, he enjoyed, on the other hand, many benfits which preponderated greatly in his favour. We do not read of mental alienation as one of

* The fierce Mamelukes were originally Tartar slaves, serving as the guards of the Ayoubite sultans. *Mamelus*, or *mameluks*, means *purchased*. They broke loose while King Louis of France was a captive in Egypt.

† Pope, the poet, has diluted Homer's vigour in the following lines:—

For any office could the slave be good,
To cleanse the fold, or help the kids to food,
If any labour those big joints could learn,
Some whey, to wash his bowels, he might earn.
To cringe, to whine, his idle hands to spread,
Is all, by which that graceless maw is fed.
Yet hear me! if thy impudence but dare
Approach yon walls, I prophesy thy fare:
Dearly, full dearly, shalt thou buy thy bread
With many a footstool thundering at thy head.—*Odyss.* 17.

Jove fixed it certain, that whatever day
Makes man a slave *takes half* his mind away.—*Ibid.* ii.

Plato, in *The Laws*, *dial.* vi., says, "Nothing in the soul of a slave is in a healthy condition." Aristotle, in his *Government* (l. i., c. 5.), agrees with Plato in advising a mixture of slaves from all countries.

the misfortunes incidental to their lot. Their occasional outbreaks were, in general, well conceived; nor do they betray any deficiency of intellect, moral courage, and foresight. Servitude is the surest discipline there is for preserving the senses against the seductions of folly. The round of daily duties, the routine of a family, the impropriety of giving vent to private feelings, or of divulging personal views and plans, do, for the most part, restrain the servant within his appointed sphere of action and capacity. His duties may curtail the dangerous sentiments of ambition and honour; but they impose the necessity of self-control, and establish the more solid virtues of prudence, integrity, and reserve, instead. It is easier to be governed than to govern; and, fortunately for the peace of mankind, the governing minds are really so few, that the multitudes cheerfully consent to obey, simply because they feel themselves to be too feeble to command.

Having sketched out the mental condition of the inferior class of the pagan world, let us raise our eyes, and examine the psychology of the higher. There was no middle class, such as we have at the present day, comprising the chief talent, wealth, and independence of society. At least, such a class was not only not numerous, but so exceedingly rare, that it scarcely ever appears on the surface. The *studio recolens* belonged to Elysium, or the schools; and the *recubans sub tegmine fagi* was nothing more than an elegant poetic ideal. The eulogies bestowed by Horace on his Sabine farm, and his delightful descriptions of rural retirement compared with the noise and smoke and bustle of the great metropolis (*fumum et opes strepitumque Romæ*), which was a singular virtue in him, are, among us, the leading propensities and the common habits of a great many persons. We have no account of *independent* folks leading a quiet life in the bosom of their families, or in modest seclusion by themselves—apart from grandeur, above want, and content with moderate means. This is a social phenomenon in the psychology of our age which we cannot value at its full worth, until we look back and analyse the "*family*" of that distant period. It was high and mighty, or it was—*nothing*. When Julius Atticus laid the immense fortune that he had accidentally discovered beneath an old family house, at the feet of the equitable Nerva, he professed to the emperor that it was too considerable for a subject, and that he knew not how to *use it*. "*Abuse it, then*," was the laconic, if not the peevish, reply of the good-natured monarch. His son, Herodes Atticus, was an instance of the prodigious wealth of the upper class. Among other magnificent works, he constructed a stadium, six hundred feet long, at Athens, built entirely of white marble, capable of admitting the whole body of the people, and finished in four years. Horace, in an ode (*non ebur neque aureum*), condemns the luxury and avarice of his countrymen, and tartly remarks, that regardless of death they built for eternity, and by their solid piers provoked the ocean as if it were never tempestuous. The stupendous bridge of Alcantara was thrown over the Tagus by a *few* Lusitanian communities. The senators of Rome and the provinces vied with each other in adorning their age and country. The golden palace of Nero, the baths of Dioclesian, and the forum of Trajan, are known to every one. But, be it remembered, all these sumptuous edifices were raised by the hands of the slaves.

There must have been some moral peculiarity in the constitution of those times, which would account for this positive division of every population on earth into the two widely separated classes of the abject slave and the overbearing man of wealth. One thing strikes us in our retrospect, which is, that polytheism nowhere existed apart from slavery. Orientals, as well as Greeks, entertained the theory of the right divine of one class over another; that some were by nature slaves, while others were kings, nobles, warriors, philosophers, &c., by birth. It was a pagan dogma inherent in their religion. Among the gods there were slaves: the Titans, the Cyclops, the Telchines, whom Jupiter destroyed by a deluge, and the obscene Cabiri of Phœnicia, who were a sort of celestial blacksmiths employed in furbishing up the metals for Vulcan, and repairing the waste and wear of the visible universe. It was the prevailing idea of paganism—an article of faith in their creed. Consequently, the slave had no ground in common for parley with the hearts of men already entrenched behind the hostile habits and concurrent attestations of antiquity. It was a state of things as old as mankind, and, moreover, rendered unassailable by precepts of supernal authority. The gods set the example. There were slaves above—*à fortiori*, there ought to be slaves below. The Cyclops in hell, and the rower who toiled at the oar along the Tiber, the Euphrates, or the Nile, were equally slaves, the only difference between them being the mythological mortality and immortality of the one and the other. It was an idea gross in the extreme, but so inveterately embodied and imbruted in the order of things, as they then were, that nothing but a divine interposition (the Gospel), or a political convulsion (the Goths), could ever have effected a permanent change in the minds of men. It was one of those rare dilemmas, to which the aphorism of the critic was exactly applicable: *Nec Deus intersit, nisi dignus vindice nodus inciderit.*

Besides this universal sentiment, there was a particular reason of state, by which the ancient governments were actuated in their policy—it was that of terror. The multitudes were governed by fear, and fear was represented by their lictors, cohorts, legions, armed triremes, the Macedonian phalanx, and the golden invincibles or body-guard of the Persian.

monarchs. In matters of superstition, likewise, *Pallor* and *Pavor* were as old as Hostilius. A sacred horror was made to thrill throughout the nerves. In the ancient bas-reliefs, this dreadful visage may still be seen, sculptured with staring eyes, mouth aghast, and hair on end—the vulgar phantom of a pantomime, in the present day. There were augurs attached to the army; and the general who formally inspected the reeking entrails of the victim just slaughtered for sacrifice, or watched the accidental flight of birds to the left hand instead of to the right, prudently shuddered at the presage of a sinister omen on the morning of a pitched battle. It was a craintive timidity of this sort that congealed the blood in the veins of the plebeian crowd, in the very heart of the greatest city of the old world, replete with its mysterious traditions, from the wolf that suckled Romulus down to the handful of thunderbolts grasped by the terrible Capitoline Jove. It has been acutely remarked, that the reign of the gods *Pavor* and *Pallor*, was the golden age of the Roman aristocracy. The proletarii dared not face the divine patricians.

Imperial Rome may be regarded as the culminating point of ancient manners. In it paganism had reached the utmost length of its projectile force, and the extreme verge of its durability. It could not accomplish anything further. It had reduced itself to a *caput mortuum* by an analytic exhaustion of the first principles of its existence. It sublimed itself within its own crucible, and remained fixed and impenetrable in that exalted position. Consequently, it is at this point of time that we are enabled to make out the psychological phenomena of paganism at the highest degree of its intensity.

In subduing the nations of the world to the beck of her iron sway, Rome invariably exerted her power in fostering slavery as the chief ingredient of her strength, under the semblance of conferring the most distinguished honours on the vanquished. The first thing she did was to enslave the gods of the conquered people, to make them her own, and to bring them captive into Italy. There was some hidden superstition, besides political craft, mixed up with this adroit proceeding; for her most gifted statesmen seem to have had an instinctive fear towards those idols of which they knew the least. Their religion betrayed its shallowness on most great occasions. The new gods were imported into the capitol; the new people followed their gods thither; they were incorporated with those of the city; and they became the slaves of Rome, or purchased their freedom at an immense cost. *Visne Romam ire, Juno ?*—to which, of course, the goddess answered in the affirmative by the monosyllable *Volo*—(*vocem quoque dicentis, Volo, auditam*). Such was the colloquy (serious or jocular, *spiritu divino, seu juvenali joco*) reported by Livy when the city of the Veii was besieged and taken by the dictator Camillus; and such was the solemn farce performed by every consul, in every quarter of the globe, before bestowing on some unfortunate set of men the unenviable title of Roman citizens.

It was the same with the pagan philosophy. Each philosopher was a petty tyrant at the head of his own narrow sect; the disciples were, during their pupilage, the slaves of their several masters; and the external world, consisting of nothing but the actual slaves of the state, was beyond the pale of civilization, instruction, sympathy, and intercommunion of thought. Intelligence was respectably lodged within the portico of the academy, the courts of law, the private mansion, and the palatial residence. The fashionable philosophy of the day strove to render itself an autocrat, and, with the egotism proper to pedantry, voted unscrupulously in favour of its own supremacy. There was no compensating force, such as the public press, or an acknowledged standard of moral rectitude, ever ready and eager to correct its erratic tendencies, to reduce it to its just proportions, and to restrain it within the circle of its proper centripetal progression. Moreover, a false quietism was the essence of each philosophic sect—sceptics, stoics, epicureans, &c., all tended to this end, which was one of ideal repose apart from the herd of men, and unconcerned in, if not indifferent to, the contests, the passions, the well-being, and the practical affairs of those around them. It was in philosophy what the statues of Phidias were in marble—a sublime immutability—a fascinating nonentity—an unreal mockery, a mental phenomenon by no means uncommon as a symptom of mania—a fixed immovable idea, incapacitating its helpless possessor for taking part in the ever-moving business of the world. Towards this kind of artificial repose the pagan philosophy tended, and it was attained by some of its most earnest followers. We behold it in its highest development in the ancient gymnosophists—the modern fakirs of India.* But it was this vicious aim and drift that ruined or neutralized its effects; for it proved itself an impracticability among the servile class, and nothing better than a barren idea in the saloons of the great, upon the couches of the luxurious, within the cabinet of the statesman, or during the service of an active campaign. It was an exotic that perished in the open air. It passed away with its professors. It propounded no scheme of education; enlightened no

* The apparently incredible things the Greeks related more than two thousand years ago, respecting the recluses of India, or *Gymnosophists*, as they called those Yogis, are found to exist even at the present day; and ocular experience has fully corroborated the truth of their narratives. There is no high conception in this department of metaphysics unknown to the Hindoos. This absorption of all thought and of all consciousness in God—this solitary, enduring feeling of internal and eternal union with the Deity, they have carried to a pitch and extreme that may almost be called a moral and intellectual self-annihilation. It is the same philosophy as that which among us has received the name of *mysticism*.—*F. Schlegel.*

popular ignorance. It dazzled a few wits with its vain pretensions; deserted its disciples in the moment of death or peril; and retired to the voluminous libraries of the learned, where it still continues to slumber in the venerable dust of ages.

The spirit of the pagan world was, in truth, the spirit of destruction. In building up the last great empire of the earth, they destroyed everything they met with. Life and death was nothing in their hands. But, as it were by a sort of judicial blindness, they did not perceive, that, in amalgamating so many diverse nations into one great dominion, they had not amalgamated, nor subdued, nor destroyed the souls of men. Practical materialists in action, they confounded the supernatural soul of man with the fortifications, the palaces, the fields, the mountains, and the rivers, in the midst of which he dwells. Real and uncompromising tyrants, they fancied that, in rivetting the chains of slavery around their conquered hosts, they had bound up their free wills in the fetters together with their limbs. They could not understand that a senatus consultum and an armed force might coerce, for a time, but never could change the affections of an injured multitude; and even the victorious Scipio started with fear or surprise, when, on his return to Rome, he met in the forum some of those whom he thought he had already extirpated by the edge of the sword in Africa. If this story be authentic, his sagacity was correct. But without the help of an anecdote, it is apparent, that *tot gentes externæ, tam sevæ*, or, at all events, those who survived *extra terminos* became, in subsequent centuries, the bitterest and most successful foes of the empire. Marcus Aurelius well-nigh perished with all his army, in an awkward military position by far too much advanced among the Germans, who were never subdued; and Augustus Cæsar once sent some legions into the depths of Arabia Deserta, where they must have been swallowed up in the sands and lost, since they never came back.

When Heliogabalus, at the height of his folly, wished to be adored as the sun, he only expressed aloud, perhaps imprudently, the secret sentiments of that political egotism which had actuated the councils of the empire, the republic, the senate, the camp, the consuls, and the kings of Rome, from the days of Numa Pompilius down to those of Honorius, the last of the Cæsars, who ended his puerile days along with his chickens within the fortress of Ravenna. As the republic came to a close, a new principle (*magnus ab integro sèclorum nascitur ordo*) insinuated itself with great subtilty into the operations of the only government on earth. The Cæsars, however abandoned they might be in their private morals, were not unfrequently high-minded in their official capacities. We find them checking the patrician barbarities, so cuttingly pointed out by Tacitus. It was by their edicts, the freedman, the miner, and the slave, obtained several privileges. Octavius protected the female,—Tiberius restrained usury. Nero, according to Suetonius, proposed gratuitous justice, and Tacitus intimates that he thought of abolishing the imposts.* The cruel Domitian, and the imbecile Claudius, vindicated both the freedom and life of the slave, and others. Hadrian, Commodus, and Alexander, protected them from personal injury and insult; and Caracalla reiterated, under his sign manual, that no one might take away that which he could never restore; and condemned perpetual bondage. He surpasses the Gracchi in his notions of equality among all *in orbe Romano qui sunt.* And Constantine made a law, by which not even sixty years' service could deprive an innocent person of his liberty. For once, in the course of ages, Paganism and the Gospel kissed each other—*venturo lætentur ut omnia sæclo.*†

Such was the gradual advance of the human mind towards better and fairer prospects. We might have presumed that its progress would have been uninterrupted, and that in another lustrum of centuries, or more, mankind would have arrived at comparative perfection and peace. But such a happy issue of events was not in the order of Providence. A deep moral evil had sapped the life of the pagan world. It could not, under the most advantageous circumstances, remodel and reform itself. In the attempt, it loosened the foundations of the state, and the aged fabric instantly tottered above their heads, fell, and buried everything beneath its ruins. Julian, Symmachus, and Zosimus, all of them pagans of the old school, had clearly foreseen and predicted the impending catastrophe. They made a last effort to prop up and secure the decayed constitution; and they succeeded for a while. Their insight was clear and penetrating; but a change had passed across the spirit of the times. The world was moving rapidly forwards; while they stood still. It passed beyond them, and they perished in its rear. Paganism died a natural death, and it was impossible to resuscitate its empty remains.

We do not know the meaning of the word psychology, if it do not embrace that of epochs as well as of individuals. In the preceding

* We have availed ourselves of the ingenious and interesting work by E. Quinet, *Le Génie des Religions*, Paris, 1851. We have not been able to verify all the references. The title does not designate the work.

† Slavery continued in force until after the Crusades, which effected such a total change in the habits, morals, politics, and prospects of the great European family. These singular campaigns, or rather flights of chivalry, were the turning point in the process of modern civilization. But for them, we should have been Mahometans, instead of Christians, at the present moment; the female character would never have obtained its proper dignity, independence, and respect, and the feudal seigneur would still have disdained to share his mess of potage with the serf. In 1135, Lothard II. had already designated, at Mayence, the *familiæ*, or nobles, the *liberi*, or *francs-bourgeois*, and the *cives opifices*, or *bourgeois-artisans*. This was a great step forward in the right direction.

pages, we have recounted succinctly the psychological phenomena of one of the most eventful periods of history. If ever there was a point of time when the accumulated experience of ages, converging in so many different radii, met, and centred itself in one single focus of power, wealth, and affluence, it was surely then. The Augustan period is the proverb of literature. The sceptre of its government is synonymous with unfailing precision and success. Its extent was co-equal with the known geography of the globe—*usque ad teminos orbis terrarum;* and it derived every benefit of arts, arms, letters, philosophy, architecture, and science, from Athens, Memphis, Ecbatana, Babylon, and Jerusalem, its conquered tributaries. With one fatal exception,—that people died there the same as elsewhere,—everything prospered at Rome.* It was all that could be achieved on earth, fifteen centuries ago; and yet this invincible, sublime, ideal ALL was shivered at a blow by the battle-axe of a barbarian; the scene was shifted; the old world passed away, and the middle ages began. The mental constitution, or the psychology, of this interesting era, it has been our endeavour to portray, and the lesson that may be learnt from it, we leave to the taste and good feelings of the ingenuous reader.

---

### Art. III.—NERVOUS INFLUENCE.†

* This remark was made by a Persian ambassador to one of the emperors during a procession through the streets of Rome. But we have mislaid our reference, and forget both the names and date. It is mentioned in Winstanley; *Eccles. and Civil History of the First Three Centuries*, vols. ii. London: 1846. A clever compendium, without an index.

